# Comparison of the Peripheral Reactive Hyperemia Index with Myocardial Perfusion Reserve by ^82^Rb PET/CT in HIV-Infected Patients

**DOI:** 10.3390/diagnostics7020031

**Published:** 2017-05-31

**Authors:** Mathilde Ørbæk, Philip Hasbak, Rasmus Sejersten Ripa, Andreas Kjær, Anne-Mette Lebech, Andreas Knudsen

**Affiliations:** 1 Department of Infectious Diseases, Copenhagen University Hospital, Hvidovre 2650, Denmark; Mathilde.jensen@sund.ku.dk (M.Ø.); lebech@dadlnet.dk (A.-M.L.); 2 Department of Clinical Physiology, Nuclear Medicine & PET, and Cluster for Molecular Imaging, Copenhagen University Hospital, Rigshospitalet and University of Copenhagen, Copenhagen 2100, Denmark; philip.hasbak@regionh.dk (P.H.); rasmus.ripa@regionh.dk (R.S.R.); akjaer@sund.ku.dk (A.K.)

**Keywords:** reactive hyperemia index, HIV, cardiovascular risk, myocardial perfusion reserve

## Abstract

After the introduction of antiretroviral therapy (ART) the life expectancy of patients infected with human immunodeficiency virus (HIV) is now approaching that of the general population and the importance of non-AIDS co-morbidities is increasing. Specifically, the risk of coronary artery disease (CAD) seems to be higher in HIV-infected patients and an accurate risk prediction of CAD is of high importance for optimal long term treatment. In this study, we assessed the correlation of the endoPAT, which is an office-based CVD screening tool with the myocardial perfusion reserve by ^82^-rubidium PET/CT. We measured the reactive hyperemia index, which is a measure of the endothelial responsiveness, by the use of an endoPAT device (Itamar Medical, Caesarea, Israel) in 48 ART treated HIV-infected patients with high CD 4 cell counts and viral suppression (HIV-RNA < 20 copies/mL), who had previously undergone measurement of the myocardial perfusion reserve by ^82^-rubidium PET/CT for study purposes. We found an inverse correlation between the reactive hyperemia index and the myocardial perfusion reserve which most likely indicates different vascular physiology. This study did not find evidence to suggest the immediate implementation of the reactive hyperemia index as a screening tool for early coronary artery disease in well-treated HIV-infected patients pending further validation in larger prospective studies.

## 1. Introduction

After the introduction of antiretroviral therapy (ART), the life expectancy of patients infected with human immunodeficiency virus (HIV) is now approaching that of the general population [1,2] and the importance of non-AIDS co-morbidities is increasing [3]. Specifically, the risk of coronary artery disease (CAD) seems to be higher in HIV-infected patients [4,5,6]. The pathogenesis behind this increased risk is not fully understood but seems to involve not only modifiable traditional risk factors, such as hypertension and smoking, but also more subtle immunological changes related to the chronic infection [7,8]. These factors seem to have potential influence on the vasculature of some HIV-infected patients [9,10,11] and accurate, easy risk prediction of CAD is of high importance for optimal long term treatment. Very early in the development towards fulminant, morphological cardiovascular disease the endothelium becomes activated/dysfunctional and plays a pivotal role in the development of atherosclerotic lesions and, as such, endothelial dysfunction has been shown to predict future cardiovascular events [12,13]. In theory, endothelial function can be investigated in any artery of the body since the atherosclerotic process appears to be universal [14]. The most commonly used method in clinical research is the flow mediated dilation of the brachial artery (FMD) [15,16]. In HIV-infected patients, it has repeatedly been found that the FMD is compromised, indicating a higher risk of cardiovascular disease than in the uninfected population [17,18]. More recently, the endoPAT has been developed which measures the reactive hyperemic response to transient arterial occlusion of the digital blood flow. This method is easier to perform and has the contralateral arm as internal control [19]. A decreased reactive hyperemic index (RHI) has been shown to predict cardiovascular events in different populations with no apparent heart disease [20,21], but to our knowledge has never been assessed in HIV-infected patients with full viral suppression. We therefore conducted a study of HIV-infected patients comparing the RHI with the vasodilator function of the coronary circulation as previously assessed by ^82^-rubidium PET/CT. This method enables the quantification of the absolute myocardial perfusion by injection of a perfusion positron-emitting tracer which allows for the estimation of the vasodilator function of the coronary circulation. Coronary microvascular dysfunction reflects the initiation and early changes in the progression toward CAD [22,23]. Indeed, ^82^-rubidium PET/CT has been found highly predictive of future cardiac events in patients with suspected CAD [24,25] and patients with diabetes or chronic renal disease with no CAD [26,27] and is considered the gold standard for assessment of coronary microvascular function [28].

## 2. Materials and Methods

### 2.1. Study Population

The participants were recruited from two previous studies described elsewhere [29,30]. In brief, the first study was a cross-sectional study comparing HIV-infected patients with HIV-uninfected controls with ^82^-rubidium PET/CT [29], and the second study was a prospective cohort study of HIV-infected women in which 44 had undergone ^82^-rubidium PET/CT [30]. Patients were asked to participate in the endoPAT study either by e-mail or included at routine visit at the out-patient HIV clinic. Exclusion criteria were asthma, pregnancy, or alcohol or drug abuse hampering the ability to adhere to the protocol. All participants were ≥18 years of age and were receiving ART.

### 2.2. Ethics

All participants received oral and written information and gave written consent before inclusion. The study was approved by the scientific ethics committee of the capital region of Denmark [H-C-2008-060, 28 July 2015].

### 2.3. Data Collection

#### 2.3.1. EndoPAT

All participants were caffeine and nicotine abstinent for 6 h prior to the examination. The EndoPAT (Itamar Medical, Caesarea, Israel) was performed according to the manufacturer’s instruction in a single session with a total duration of approximately 30 min including 10 min of rest, 5 min of total occlusion of the brachial arteries, and 10 min post-occlusion rest. Blood pressures were measured prior to the test. The ratio between rest and post-occlusion was calculated automatically by the EndoPAT software, providing the reactive hyperemia index (RHI). All measurements were performed by a single observer. The mean duration between the ^82^-rubidium PET/CT and endoPAT studies was 3.3 years (range 2.0–4.3).

#### 2.3.2. PET Imaging

All patients in this study had previously undergone ^82^-rubidium PET/CT for study purposes and the methods have been described in detail elsewhere [29]. In brief, patients were scanned in a single session on a Siemens Biograph mCT/PET 128-slice scanner (Siemens Helthcare, Knoxville, TN, USA) during rest and stress after injection of ^82^-rubidium supplied from a CardioGen-82 Sr-82/Rb-82 generator (Bracco Diagnostics Inc., Princeton, NJ, USA). Stress images were acquired during the infusion of adenosine. The myocardial perfusion reserve was calculated as the myocardial stress perfusion/myocardial rest perfusion. These values were corrected for baseline cardiac work [31]. A low-dose CT was performed for attenuation correction. Coronary artery calcium score (CACS) images were acquired from a non-contrast breath-hold CT. The CACS was calculated according to the Agatston score using a threshold of 130 Hounsfield units (HU) [32].

#### 2.3.3. Blood Markers

Blood samples were drawn at routine visits at the HIV out-patient clinic and analysed for CD 4 cell counts by flow cytometry using the BD Multitest™ (BD Biosciences, San Jose, CA, USA), and HIV RNA using the AmpliPrep/COBAS, TaqMan HIV-1 test vers. 2.0 (Roche, Branchburg, NJ, USA). Quantitative determination of blood lipids was performed using the Cholesterol gen2 (Roche Diagnostics, GmBh). All blood tests were routinely performed by the hospital laboratory.

#### 2.3.4. Risk Score

The Framingham risk score (FRS) was calculated according the published definitions [33] as the risk of CHD in 10 years.

### 2.4. Statistical Analyses

Data are shown as mean ± standard error of the mean (SEM), median (IQR) or number (percentage) where relevant. Correlation analyses were performed by Pearson correlation on log-transformed data, whereas groups were compared by unpaired *t*-test on log-transformed data or by analysis of variance (ANOVA) if more than two groups. Categorical variables were compared by Chi-square test.

Statistics were performed on SPSS 22 (IBM SPSS statistics for Windows, version 22.0; IBM Corp, Armonk, NY, USA).

## 3. Results

Baseline characteristics of the 48 patients are presented in Table 1. Our study included more men than women with a relatively wide age span of 37–72 years. Two of the patients had diabetes mellitus type 2, and one of these received antidiabetic medication. One of the patients had hepatitis B (defined as HBS antigen positive), whereas none had hepatitis C. The mean Framingham risk score (FRS) of developing coronary heart disease within 10 years was intermediate (10–20%) for the entire group, but the span was wide (range 0.2–42%), and 63% of the patients had a low FRS. A duration of 3.3 years was found between the quantification of the MFR and the RHI, and in that time the mean FRS rose 3.4%, which mainly can be attributed to the effect of age in the FRS algorithm.

Table 2 shows that all patients included in this study were receiving ART and they all had high CD 4 cell counts and no detectable viremia (HIV-RNA < 20 copies/mL). Table 3 shows data from the ^82^-rubidium myocardial perfusion study and the endoPAT study. In our study, 70% of the patients had a normal myocardial flow reserve as assessed by ^82^-rubidium PET/CT and 67% had a normal reactive hyperemia index.

**Figure 1:**
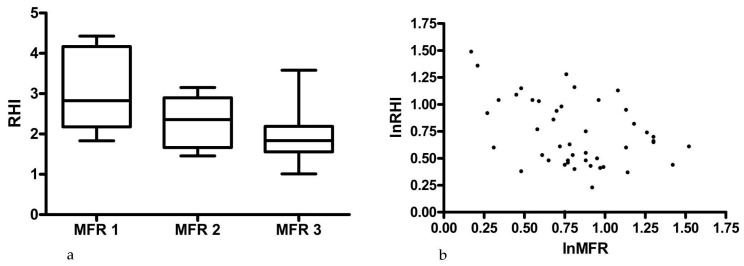
(**a**) Shows a box plot with the mean values (whiskers depict 95% confidence interval) of the three groups of MFR (MFR 1 < 1.5; MFR 2 ≥ 1.5 < 2.0; MFR 3 ≥ 2.0; (**b**) Illustrates the inverse correlation between MFR and RHI. MFR, myocardial flow reserve; RHI, reactive hyperemia index.

As can be seen in Figure 1a, the patients with normal MFR and normal RHI were not the same since a significant difference was found between the groups in the ANOVA (*p* = 0.007). Further, a negative linear correlation was found between the levels of MFR and RHI as shown in Figure 1b (*r* = −0.38, *p* = 0.009), and this linear correlation was not explained by differences in underlying CHD risk since adjustment for FRS only attenuated this correlation slightly (*r* = −0.32, *p* = 0.03). The patients with a positive CACS (defined as CACS ≥ 1) did not have impaired RHI (2.3 vs. 2.1; *p* = 0.55), and a positive correlation was found between CACS and RHI even when adjusting for FRS (*р* = 0.72, *p* = 0.02), whereas an inverse correlation was found for CACS and MFR also adjusting for FRS (*р* = −0.70, *p* = 0.01).

## 4. Discussion

In this follow-up study of 48 HIV-infected patients, who had all previously undergone ^82^-rubidium PET/CT for study purposes, we studied the correlation between the peripheral flow mediated dilation (by the so-called reactive hyperemia index using the endoPAT) and the myocardial flow reserve to assess the value of the endoPAT as a screening tool for early CAD. This method would offer an easy and non-invasive way to assess changes in coronary vasomotor function as a sign of early CAD. Contrary to our hypothesis, we found an inverse correlation between the MFR and RHI and as such we recommend that the endoPAT be validated in larger prospective studies before the implementation as a screening tool for CAD among HIV-infected patients. Endothelial dysfunction has been shown to be an early step towards CVD [34,35] and studies have shown a prognostic value of the RHI [21] as well as a discriminative power for coronary endothelial dysfunction as assessed by infusion of acetylcholine during cardiac catheterization [20]. The lack of accordance between the two assessments in this study can be explained by differences in the underlying vascular physiology. Both methods relay on endothelial derived responses, but adenosine (used in our study) and dipyridamole (another widely used vasodilating agent) act on the A2 adenosine receptor (and dipyridamole by inhibition of reuptake of endogenous adenosine) activating G-proteins, causing relaxation of the coronary circulation. On the other hand, flow mediated dilation such as the endoPAT method is mediated through effects of nitric oxide (NO) [36]. It therefore follows that studies using NO-mediated vasodilation of the coronary arteries are more likely to find a correlation between myocardial flow and reactive hyperemia response, such as studies using acetylcholine [20,37], whereas studies using the NO-independent adenosine or dipyridamole tend to report no correlation [38,39,40].

Interestingly, already one of the first studies to validate the endoPAT against myocardial perfusion found that there was no correlation using adenosine [20]. Studies of reactive hyperemia in HIV-infected patients usually use the brachial artery reactivity test and have more or less all shown a tendency towards impaired endothelial function [17,18]. However, studies comparing flow mediated dilation of the brachial artery and the circulation in the fingertip have shown that these methods provide different information [41,42] although a recent meta-analysis showed that they both have predictive value for cardiovascular events in the non-HIV-infected, general population [43] Further questioning the use of the endoPAT as a screening tool in this population was the finding of a normal RHI in patients with abnormal CACS and an positive correlation between the CACS and RHI. To our knowledge, this study is the first to compare the coronary and peripheral circulation in HIV-infected patients and our findings compare well with other studies in the general population using adenosine as myocardial stress agent, where no correlation between myocardial perfusion and peripheral hyperemia response is found in patients without signs of CAD. Our results must be interpreted in light of the small sample of patients and the fact that a high proportion had low FRS and no known CAD. The assessment of the endoPAT should therefore also be carried out in at larger cohort with higher risk of cardiovascular disease. Also, an interval of more than three years was found between the ^82^-rubidium PET/CT and the endoPAT-study and subjects in the study could have made lifestyle changes which are not accounted for here, which could possibly affect the reactive hyperemia index. However, we found a mean increase in FRS of 3.4% between the two studies, which can be explained predominantly by the increase in age. Finally, this study is cross-sectional including only HIV-infected patients without the possibility of comparison to a non-infected population.

## 5. Conclusions

In this first comparative study of the peripheral reactive hyperemia index by the endoPAT and the myocardial perfusion reserve as assessed by ^82^-rubidium PET/CT, we found an inverse correlation between the two methods. Larger prospective studies are warranted to confirm our findings before any recommendations can be made on the use of the endoPAT as an office-based screening tool for detection of early CAD among HIV-infected patients.

## Figures and Tables

**Table 1:** Baseline characteristics of the patients.

Parameters		*N* = 48 (%)
Sex		
	Male	30 (63)
	Female	18 (37)
Age, years		55 ± 1
Interscan duration (years) #		3.3 ± 0.1
Smoking		
	Active	9 (19)
	Former	21 (44)
	Never	18 (37)
Medication		
	Antihypertensive	13 (27)
	Statin	9 (19)
	Anticoagulant	8 (17)
Perfusion defects on PET/CT		3 (6)
FRS (CHD 10 years, %)		11.2 ± 1.5
ΔFRS (CHD 10 years, %) ^¤^		3.4 ± 0.9
Lipids		
	Total cholesterol, mmol/L	5.6 ± 0.2
	HDL, mmol/L	1.4 ± 0.1
	LDL, mmol/L	3.4 ± 0.1
	Triglycerids, mmol/L	1.9 ± 0.3
Systolic Blood Pressure, mmHg		132 ± 3
Diastolic Blood Pressure, mmHg		78 ± 2
BMI		24.8 ± 0.7
Diabetes mellitus		3 (6)
Blood glucose, mmol/L		6.0 ± 0.3	

Data in this table are presented as number (%) or mean ± standard error of the mean. # Duration in years between ^82^-rubidium PET/CT and endoPAT study. ^¤^ The mean increase in FRS between the ^82^-rubidium PET/C and the endoPAT study. BMI, body mass index (kg/height in m^2^); CHD, coronary heart disease; FRS, Framingham risk score; HDL, high density lipoprotein; LDL, low density lipoprotein.

**Table 2:** HIV related data.

Parameters	*N* = 48
CD 4 cell count (10^6^/L), median (IQR)	672 (528–844)
HIV RNA (copies/mL), median (IQR)	19 (19–19)
HIV duration, years, median (IQR)	18.0 (12–24)
ART duration, years, median (IQR)	16 (11–19)
ART regimens	
2 NRTI + 1 NNRTI	25 (52)
2 NRTI + PI	8 (17)
2 NRTI + PI + IH	5 (10)
Other	10 (21)

Data in this table are presented as median (IQR, interquartile range) or number (%). ART, antiretroviral therapy; IH, integrase inhibitors; NNRTI, non-nucleoside reverse transcriptase inhibitors; NRTI, nucleoside reverse transcriptase inhibitors; PI, protease inhibitors.

**Table 3:** Data from the original ^82^-rubidium study and the RHI from the present endoPAT^®^ study.

Parameters	Value
Myocardial flow reserve, mean ± SEM	2.4 ± 1.1
Myocardial flow reserve tertiles	
<1.5	11%
≥1.5 < 2.0	19%
≥2.0	70%
CACS, median (range)	0 (0–1884)
RHI, median (range)	1.9 (1.0–4.4)
Low RHI (<1.67)	33%
Normal RHI (≥1.67)	67%

CACS, coronay calcium score; RHI, reactive hyperemia index.
